# Zika virus infects human osteoclasts and blocks differentiation and bone resorption

**DOI:** 10.1080/22221751.2022.2086069

**Published:** 2022-06-14

**Authors:** Noreen Mumtaz, Marijke Koedam, Johannes P. T. M. van Leeuwen, Marion P. G. Koopmans, Bram C. J. van der Eerden, Barry Rockx

**Affiliations:** a Department of Viroscience, Erasmus University Medical Centre, Rotterdam, the Netherlands; b Department of Internal Medicine, Erasmus University Medical Centre, Rotterdam, the Netherlands

**Keywords:** Zika virus, osteoclast, differentiation, bone resorption, pathogenesis

## Abstract

Bone-related complications are commonly reported following arbovirus infection. These arboviruses are known to disturb bone-remodeling and induce inflammatory bone loss via increased activity of bone resorbing osteoclasts (OCs). We previously showed that Zika virus (ZIKV) could disturb the function of bone forming osteoblasts, but the susceptibility of OCs to ZIKV infection is not known. Here, we investigated the effect of ZIKV infection on osteoclastogenesis and report that infection of pre- and early OCs with ZIKV significantly reduced the osteoclast formation and bone resorption. Interestingly, infection of pre-OCs with a low dose ZIKV infection in the presence of flavivirus cross-reacting antibodies recapitulated the phenotype observed with a high viral dose, suggesting a role for antibody-dependent enhancement in ZIKV-associated bone pathology. In conclusion, we have characterized a primary *in vitro* model to study the role of osteoclastogenesis in ZIKV pathogenesis, which will help to identify possible new targets for developing therapeutic and preventive measures.

## Introduction

Zika virus (ZIKV) is an arthropod-borne flavivirus and is primarily transmitted by a bite of *Aedes* mosquitoes. Since the initial discovery in Uganda in 1947, an outbreak of ZIKV disease on the Island of Yap (2007) and later in Polynesia (2013), highlighted the epidemic potential of ZIKV [1,2]. ZIKV gained attention worldwide after its emergence and rapid widespread distribution in the Americas and the Caribbean in 2015 [3]. In acute ZIKV infection, symptoms like fever, rash, conjunctivitis, myalgia, and arthralgia are commonly observed. Arthralgia has been reported in over 70% of symptomatic ZIKV cases, including arthralgia persisting for more than thirty days [4,5]. ZIKV infection-associated complications were observed during pregnancy, called congenital ZIKV syndrome including microcephaly [6], and were the reason to declare ZIKV a public health emergency of international concern (PHEIC) by the World Health Organization. ZIKV associated microcephaly is widely accepted as a neurodevelopment disorder. As a consequence, extensive pathogenesis studies, mainly using neuronal models, are performed to explore the neurological link between ZIKV infection and microcephaly. In various *in vitro* studies, it has been shown that ZIKV preferentially infects brain cells, in particular, human neural progenitor cells, which can rationalize the ability of ZIKV to impair the development of the fetal brain and subsequently cause microcephaly along with other neurodevelopmental abnormalities [7–10]. However, the detection of ZIKV RNA in mesenchymal stromal cells (MSCs, precursors of bone forming cells) in the perichondrium of a fetus [11] also points to the potential effect of ZIKV infection on bone remodeling. This hypothesis was further reinforced by our previously reported study, where we demonstrated that ZIKV infection impaired osteoblast function [12].

Bone remodeling is a continuous process, which mainly depends on the concerted action of two key players of bone homeostasis, namely the bone forming osteoblasts (OBs) and the bone resorbing osteoclasts (OCs), both under the control of osteocytes [13]. Bone remodeling is affected by several microorganisms including bacteria and viruses [14]. Viruses, particularly arthropod-borne viruses such as Chikungunya virus (CHIKV), Ross River virus (RRV), and Dengue virus (DENV), exert a negative impact on bone self-repair mechanism by unbalancing bone homeostasis [15,16], resulting in joint pain. During CHIKV and RRV infections, the bone undergoes remodeling where osteoclast function is extensively activated due to an upregulation of pro-inflammatory cytokines (paracrine factors), thus favouring bone resorption and causing exacerbated bone loss [17,18].

OCs are bone tissue-specific giant multinucleated cells that derive from the monocyte/macrophage hematopoietic lineage. The formation of mature OCs is regulated by macrophage colony stimulating factor (M-CSF), and a key osteoclastogenic marker, receptor activator nuclear factor kappa B ligand (RANKL). Mononucleated precursors (MPs) fuse to form terminally differentiated OCs [19,20]. These MPs include both blood circulating monocytes and bone resident precursors. MPs are also reported as the main targets of replication for some viruses such as ZIKV [21]. For arthropod-borne flaviviruses, particularly for dengue virus (DENV) and ZIKV, bone-related pathologies are frequently reported, but the direct effect of infection on osteoclasts has only been studied for DENV, where an increased osteoclast activity is reported due to DENV infection [22–24].

Since Fcγ receptors are widely expressed on the surface of immune cells such as monocytes, macrophages, and OCs [25,26], in addition to direct infection, ZIKV can potentially infect OCs in the presence of cross-reactive antibodies and result in antibody dependent enhancement (ADE) [25]. As the geographic range of ZIKV overlaps with that of DENV, ADE has been postulated to be one of the main risk factors for complications of ZIKV infection, such as microcephaly [27].

Here, we aim to investigate the direct effects of ZIKV infection on osteoclastogenesis. We first determined the susceptibility of human peripheral blood mononuclear cells (PBMCs)-derived osteoclast precursors to ZIKV infection. We also investigated the effect of infection on osteoclast maturation and resorption activity. Next, we evaluated the susceptibility status at different stages of differentiation of OCs, and the effect on osteoclastogenesis. Our findings demonstrate that OCs at different stages of differentiation are susceptible to ZIKV infection. However, the effect on the phenotype is more evident when infected at an early stage of differentiation, especially using either a high MOI or a low MOI in the presence of pan-flavivirus cross-reacting antibodies.

## Materials and methods

### Cells

**Figure 1.**
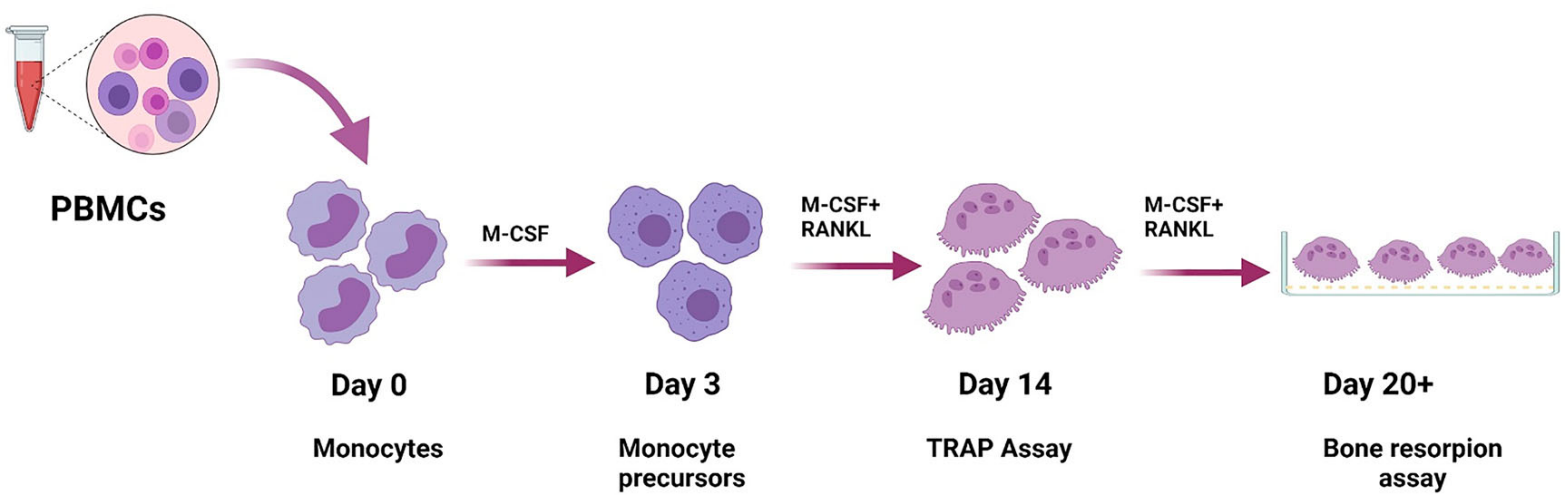
Schematic overview of PBMCs derived osteoclast culture model. Four hours following seeding, cells were rinsed twice with PBS to remove non-adherent cells and treated with M-CSF. From day 3 onward, RANKL was supplemented as well to induce osteoclastogenesis. On day 14 post seeding, TRAP staining was performed to determine the formation and differentiation of multinucleated OC. Around day 20, bone resorption assay was done to determine the resorptive activity of OCs.

Buffy coats were obtained from anonymous healthy donors (Sanquin) and PBMCs were isolated as described before [28]. Briefly, buffy coats were diluted 1:1 in phosphate buffered saline (PBS). The diluted PBMCs are layered onto lymphoprep in a 2:1 ratio. After centrifugation, PBMCs were collected from the interphase and washed twice with PBS. The cells were frozen until further use. At the time of the experiment, cells were thawed, counted, and seeded at a density of 5 × 10^5^ cells per/well in 96-well plates in α-MEM culture medium (Gibco, Thermo Fisher Scientific, Breda, The Netherlands) supplemented with 100 units/ml penicillin, 100 μg/ml streptomycin (Thermo Fisher Scientific, Breda, The Netherlands), 250 ng/ml amphotericin B (Sigma, St. Louis, MO, USA), 1.8 mM CaCl_2_ (VWR International BV), and 15% (vol/vol) heat-inactivated fetal calf serum (FCS; Thermo Fisher Scientific). Following seeding, cells were incubated for 4 h and then rinsed twice with PBS to remove non-adherent cells. Next, a culture medium was added containing 25 ng/ml human macrophage-colony stimulating factor (M-CSF; R&D Systems, Abingdon, UK). From day 3 onward, cells were cultured in the presence of both M-CSF and 30 ng/ml human receptor-activated NF-κB ligand (RANKL; PeproTech, London, UK) (Figure 1). Depending on the experiment, cells at different stages; pre-(day 3-post seeding), early (day 7-post seeding), and late (day 10-post seeding), were selected for infection. Vero cells (African green monkey kidney epithelial cells, ATCC #CCL-81) were cultured in Dulbecco’s modified Eagle’s medium (DMEM, Lonza, the Netherlands) supplemented with 10% heat-inactivated fetal bovine serum (FBS, Greiner Bio-One, Austria), 2 mM L-glutamine (Lonza), 1% sodium bicarbonate (Lonza), 1% Hepes (Lonza), 100 U/mL penicillin (Lonza) and 100 µg/mL streptomycin (Lonza) at 37°C and 5% CO_2_ in a humidified atmosphere.

### Virus

Zika virus Suriname strain ZIKVNL00013 (Asian lineage) was isolated from a female patient in the Netherlands who travelled to Suriname (ZIKVAS-Sur16, [EVAg no. 011V-01621]). The virus stock was grown in Vero cells, and passage 3 was used for the current study [7,11]. Virus titers in the supernatants, with detection limit of 10^1.5^ TCID_50_/ml, were determined as described previously [12].

### Replication kinetics of ZIKV

On day 3 post-seeding, mononuclear osteoclast precursors (pre-OCs) were infected with ZIKV at a multiplicity of infection (MOI) of 5 for an hour at 37°C in 5% CO_2_. After incubation, the supernatant was removed and cells were washed three times with αMEM medium containing 15% heat-inactivated FCS. The cells were cultured in αMEM as described above for 2–3 weeks. Uninfected controls were cultured in parallel. In addition to early-stage infection, cultures were also infected at different stages of differentiation including early (day 7) and late differentiating (day 10) OCs using MOI of 5. Moreover, pre-and early differentiating OCs were also infected with different MOIs (0.1, 1, and 5). To determine the ZIKV infectious titers produced, cell supernatants were collected at different time points post infection, and the supernatant was stored at −80°C until further use.

### Antibody dependent enhancement assay (ADE)

On day 3 post-seeding, serially diluted humanized pan-flavivirus mAb (hu4G2, Native Antigen Company, UK) was incubated with ZIKV at MOI of 0.1 (ADE-0.1) for one hour at 37°C in the humidified atmosphere before adding them to cells. After an hour of incubation, virus-antibody immune complexes were added to the cells and incubated for one hour at 37°C. Subsequently, cells were washed three times with medium and were cultured in αMEM as described above for two days. The supernatant was harvested at day 2 post-ADE and frozen at −80°C until further use. Later, virus titers were determined by endpoint titrations on Vero cells. In ADE assays, controls such as virus without antibody (MOI 0.1 & 5) and mock infections were also included.

### Immunofluorescence assay

Infected cells from the replication growth kinetics assay were fixed with 4% PFA at day 6 post-seeding, permeabilized with 70% ethanol, and stained using the mouse monoclonal antibody anti-flavivirus group antigen (MAB10216), clone D1–4G2-4-15 (Millipore, Germany) followed by staining with goat anti-mouse IgG conjugated with Alexa Fluor 488 (Life technologies, the Netherlands) in an immunofluorescence assay (IFA) as described previously [12,29]. For fusion markers, early infected and mock controls were fixed and permeabilized using 0.1% Triton X-100 at different time points. Immunofluorescent staining was performed using mouse anti- Nuclear Factor of Activated T-cells, Cytoplasmic 1 (NFATC1) antibody (Invitrogen, Thermo Fisher Scientific, USA) and rabbit anti- Transmembrane 7 Superfamily Member / Dendritic Cell-Specific Transmembrane Protein (TM7SF4/DC-STAMP) antibody (Abcam, Cambridge, UK) followed by staining with Alexa Fluor 555-conjugated goat anti-mouse IgG (Life technologies) and Alexa Flour 647-conjugated goat anti-rabbit (Life Technologies), respectively. The nuclei were labelled with DAPI (Invitrogen, Thermo Fisher Scientific, USA). Subcellular localization of NFATC1 and TM7SF4 was observed using a Zeiss LSM 700 confocal laser scanning microscope fitted onto an Axio observer Z1 inverted microscope (Zeiss, Breda, the Netherlands).

### Adhesion assay

On day 10 post-seeding, an adhesion assay was performed with early infected OCs and mock-infected controls. The supernatant was removed and cells were washed twice with PBS. Then cells were incubated with accutase to detach the cells (Merck Live Science, BV) for 10 min at 37°C. After incubation, cells were washed with PBS, adherent cells were fixed with 4% formalin, and nuclei were stained with DAPI (Invitrogen, Thermo Fisher Scientific, USA). Five images per well were taken and the surface area of adhered nuclei was quantified (Axiovert 200, Zeiss). All images were processed using Image J software (version 1.47).

### TRAP staining

On day 14 post-seeding, cells were fixed with 10% formalin and stained, using a tartrate resistant acid phosphatase (TRAP) leucocyte kit (Sigma, St. Louis, MO, USA) as described previously [30]. To limit the variation between wells, five images per well were taken, using an Axiovert 200 microscope (Zeiss). The number of OCs and nuclei per osteoclast were counted by Image J software (https://imagej.nih.gov/ij/). The fusion index (%) was calculated as the total number of nuclei in TRAP-positive multinucleated cells (>5) divided by the total number of nuclei counted*100.

### Bone resorption assay

To assess the mineral resorption by OCs, pre-OCs were cultured on osteoassay surface plates (Corning, NY, USA) for 18–21 days and then von Kossa staining was performed [30]. The supernatant was removed and cells were washed with water. After washing, wells were stained for 30 min with 5% silver nitrate (in bright daylight), incubated for one minute in 5% sodium carbonate in 25% formalin, and finally for 2 min in 5% sodium thiosulphate. Pictures were obtained using an Axiovert 200 (Zeiss). Areas that have been resorbed by functional OCs appear as white pits, whereas the non-resorbed sliver-stained mineral is black. The percentages of the resorbed area by differentiated OCs were quantified using Image J software (version 1.47).

### Phalloidin staining protocol

To analyze the effect of infection on actin rings, phalloidin staining was performed on different time points post-seeding (day 10, day 14, and day 18) based on the appearance of mature OCs in mock-infected controls. Infected and mock-infected cultures were washed with PBS and fixed with 10% formalin. The staining procedure was described previously [31]. Shortly, PBS + 0.1% Triton-X100 was added for 10 min, followed by PBS + 0.05% Tween and 1% BSA for 30 min. Cells were then incubated with rhodamine-conjugated phalloidin antibody (Thermo Fisher Scientific) for 1 h at RT and washed with PBS + 0.05% Tween followed by DAPI staining. Staining of the cytoskeleton was visualized by using a Zeiss LSM 700 confocal laser scanning microscope fitted onto an Axio observer Z1 inverted microscope (Zeiss). All images were processed using Image J software (version 1.47).

### Quantitative real-time PCR analyses

The methods used for RNA extraction, cDNA synthesis, and RT–PCR have been described previously [32]. Real-time qPCR was performed using the QuantStudio™ 7 Flex Real-Time PCR System (Applied Biosystems), and the results were analyzed using QuantStudio TM Real-Time PCR software v1.3 (Applied Biosystems). Data are presented as relative mRNA levels calculated by the formula: 2^−Δ (Ct of gene of interest − Ct of housekeeping gene)^. All primer sequences used are summarized in Table 1.

**Table 1.** Primer sequences of the analyzed genes.

Gene	Forward primer (5′-3′)	Reverse primer (5′-3′)
GAPDH	CCGCATCTTCTTTTGCGTCG	CCCAATACGACCAAATCCGTTG
ACP5	CTGCCTACCTGTGCGGCCAC	CTCAGCACGTAGCCCACGCC
FOS	CCAGCATGGGCTCGCCTGTC	CGGCCAGGTCCGTGCAGAAG
CTSK	TGCCCACACTTTGCTGCCGA	GCAGCAGAACCTTGAGCCCCC
OSCAR	CACTCCGTCTGTGGCCATTA	CCAGGGGTCACAACTGTAGC
ATPV0D2	TTCTTGAGTTTGAGGCCGACA	AGAGTTTGCCGAAGGTTGGA

ACP5, Acid Phosphatase 5; FOS, Fos Proto-Oncogene GAPDH, Glyceraldehyde 3-phosphate dehydrogenase; OSCAR, human osteoclast-associated receptor; TRAP, Tartarate-resistant acid phosphatase.

### Statistics

The statistical analyses were performed using GraphPad Prism 5.01 software. All results are expressed as means with standard error of the mean (SEM). Unpaired t-test was used to compare infected versus un-infected conditions, *p* value ≤ 0.05. For comparison of multiple data sets, Kruskal–Wallis test (one-way ANOVA test, non-parametric) and Dunnett’s multiple comparison test was performed, *p* value ≤ 0.05.

## Results

### Osteoclast precursors are susceptible to ZIKV infection

**Figure 2.**
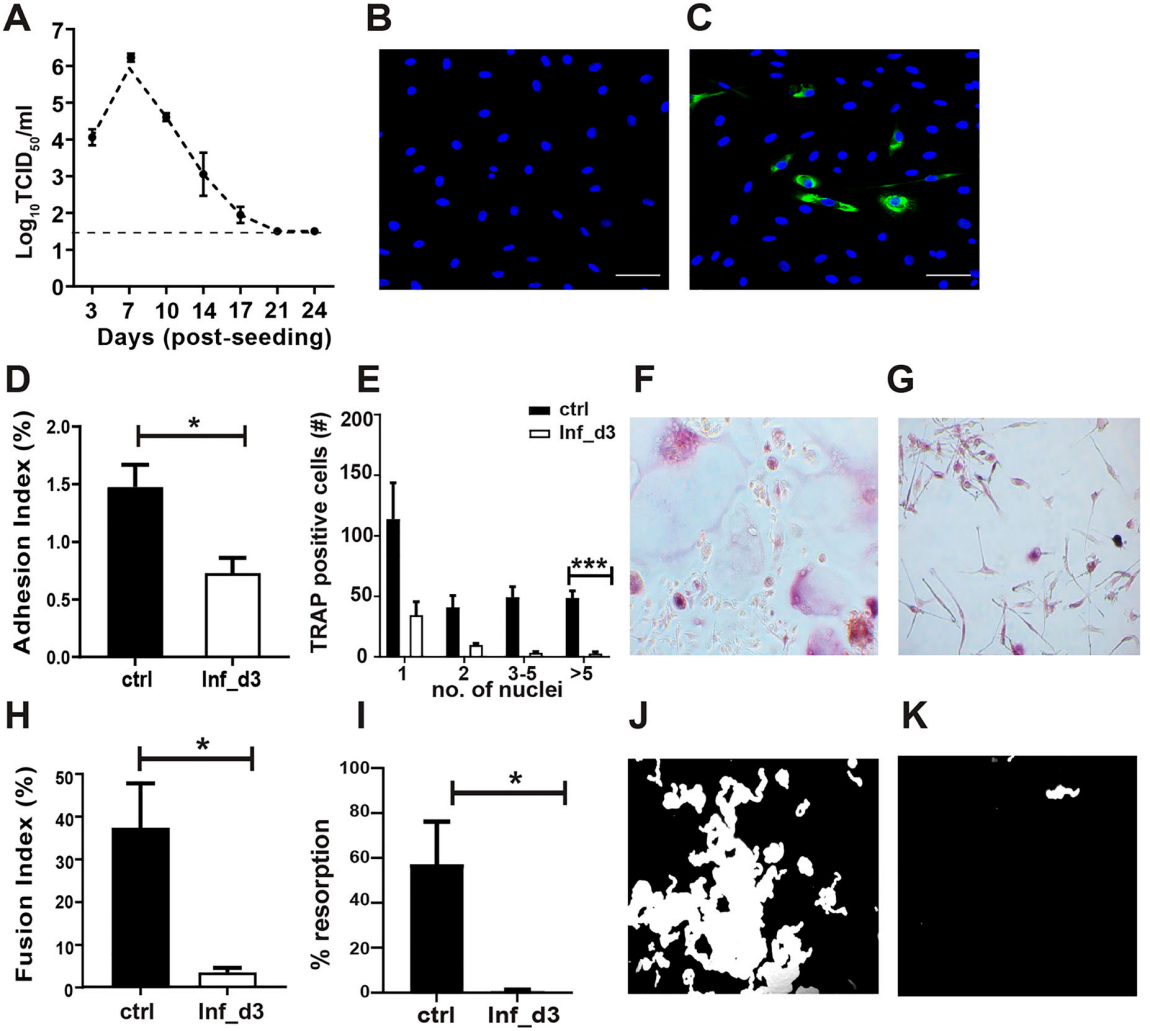
Replication of ZIKV in pre-OCs infected at day 3 post seeding. Culture supernatant was collected twice a week for the period of three weeks. (**A**) Growth curve kinetics of ZIKV infection in pre-OCs after infection with 5 MOI. (**B**) Representative immunofluorescent images of uninfected controls and (**C**) ZIKV-infected cells stained for ZIKV antigen (green) and nuclei (blue) on day 2 post-infection (dpi). Magnification 200x. (**D**) On day 10 post cell culturing, adhesion index is calculated by measuring the area adhered by remaining nuclei after Accutase treatment. (**E**) On day 14 post cell culturing, effect of infection on OC formation is measured by quantifying the number of nuclei in TRAP + cells, (**F**) representative images of TRAP-stained mock infected controls and (**G**) ZIKV infected pre-OCs. (**H**) On day 14 post cell culturing fusion index is also measured by counting the total number of nuclei in TRAP-positive multinucleate cells (>5) divided by total number of nuclei counted*100 (**I**) On day 21 post cell culturing, resorption function is assessed by quantifying the resorption pits, (**J**) representative images of von Kossa staining for mock infected controls and (**K**) ZIKV infected pre-OCs. Results are compared between ZIKV infected (white bars) and mock infected controls (black bars). *N* = 3 for replication kinetics, *N* = 4 for adhesion assay, *N* = 5 for TRAP assay and fusion index, *N* = 4 for resorption assay. Error bars represent SEM. * *p* < 0.05

To determine the susceptibility of osteoclast precursors to ZIKV infection, we infected the pre-OCs with ZIKV at an MOI of 5. We determined replication kinetics for three weeks and found that pre-OCs were infected with ZIKV. Peak titers up to 10^6.5^ TCID_50_/ml were observed on day 3 post-infection and shedding of virions dropped over the subsequent time period studied, but remained detectable (Figure 2A). Following infection, we did not observe any evidence of cytopathic effects (CPE) during the culture period (supplementary Figure 1), although altered morphology, such as stretched fibroblast-like cells, was observed. Infection of OCs was also confirmed by IFA (Figure 2B and C).

### ZIKV-infected pre-OC have reduced adhesion ability

The adhesion ability is crucial for effective bone resorption, therefore we investigated the adhesion of ZIKV-infected OCs and observed a significant decrease (>2 fold, *p* = 0.01) in the adhesion index of ZIKV-infected pre-OCs on day 7 post-infection (Figure 2D).

### Early ZIKV infection leads to reduced TRAP positive mature OC number and fusion index

To identify the effect of early infection on OCs differentiation, we performed a TRAP assay on day 14 post-seeding and counted the number of nuclei in TRAP positive OCs. Cells with >5 nuclei were categorized as mature OCs. In early infected pre-OCs, there was a significant reduction (≈45 fold, *p* =  3 × 10^−4^) in the number of mature multinucleated OCs compared to mock-infected controls (Figure 2E–G). To measure fusion efficacy, we also determined the fusion index in ZIKV-infected pre-OCs versus mock-infected controls and observed a significant reduction (≈11 fold, *p* = 0.03) in the fusion index in ZIKV-infected cultures (Figure 2H).

### ZIKV-infected pre-OC have reduced resorption

To determine the resorption ability and functionality of ZIKV infected pre-OCs, we performed von Kossa stainings. The resorption function was significantly reduced (≈67-fold, *p* =  0.02) in ZIKV-infected cultures compared to mock-infected controls (Figure 2I–K).

### ZIKV infection-induced effect on osteoclastogenesis is stage dependent

**Figure 3.**
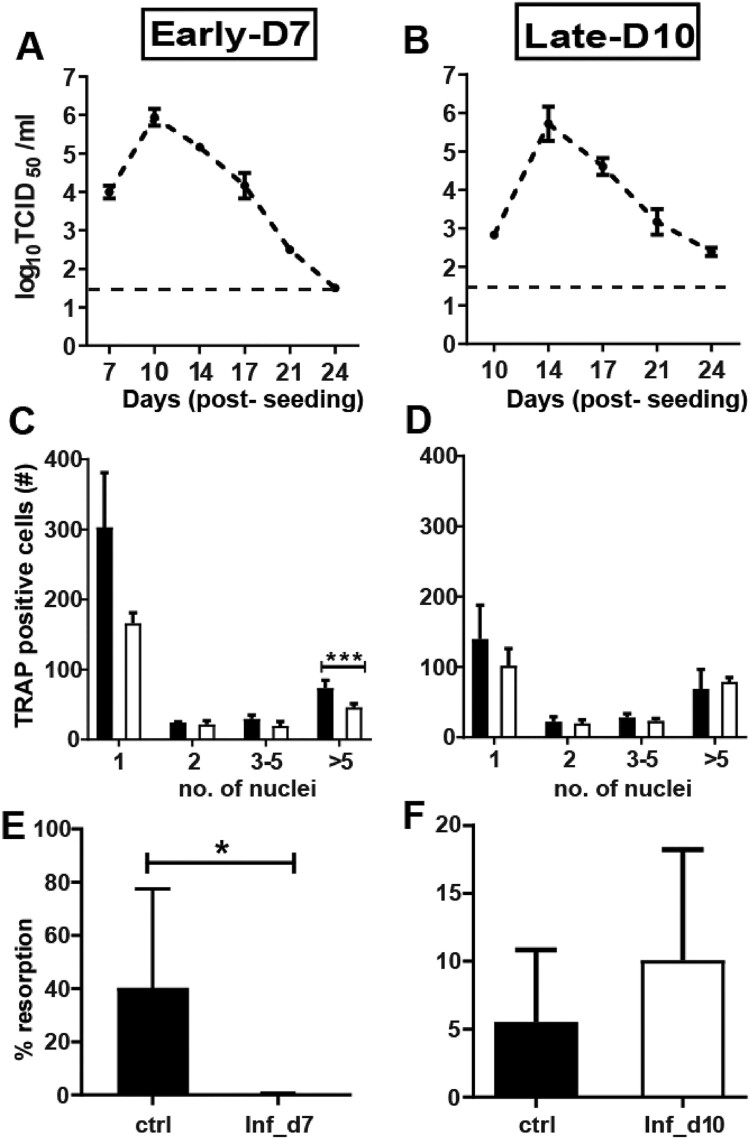
Replication of ZIKV in OCs infected at different stages of differentiation. (**A–C**) Growth curve kinetics of ZIKV infection in (**A**) early differentiating (Inf_d7) and (**B**) late differentiating (Inf_d10) OCs after infection using MOI of 5. (**C-D**) TRAP staining is performed on day 14 post cell culturing in ZIKV infected (**C**) early differentiating (Inf_d7), (**D**) late differentiating (Inf_d10) OCs and mock infected controls. (**E-F**) Von Kossa staining is performed on day 20 post cell culturing in (**E**) early differentiating OCs and (**F**) late differentiating OCs. Results are compared between ZIKV infected (white bars) and mock infected controls (black bars) for each infection time point. *N* = 3 for replication kinetics, *N* = 5 for TRAP assay, *N* = 4 for resorption assay. Error bars represent SEM. * *p* < 0.05.

To evaluate the susceptibility of osteoclasts at different stages during differentiation, the cells were differentiated to early and late differentiating OCs and infected with ZIKV. Similar to ZIKV infected pre-OCs (Figure 2A), we observed that OCs in early and late stages of differentiation were equally susceptible to infection. Peak titers and kinetics were similar across differentiation stages (Figure 3A and B). We subsequently investigated the effect of ZIKV infection on the differentiation and function of differentiating OCs (Figure 3C–F). Despite the similar susceptibility to infection, a reduction in differentiation (*p* = −4 × 10^−4^) and resorption (*p* = −0.03) was only seen in ZIKV infected early-stage OCs (Figure 3C and E). In day 10 infected OCs (late stage), contrary to ZIKV infected pre-and early stages of OCs (Figures 2D and 3C, respectively), we did not observe any difference in OCs formation between infected and control groups (Figure 3D). Likewise, infection on day 10 post-seeding did not affect the resorption capacity (Figure 3F).

### ZIKV infection affects OCs in a dose-dependent manner

**Figure 4.**
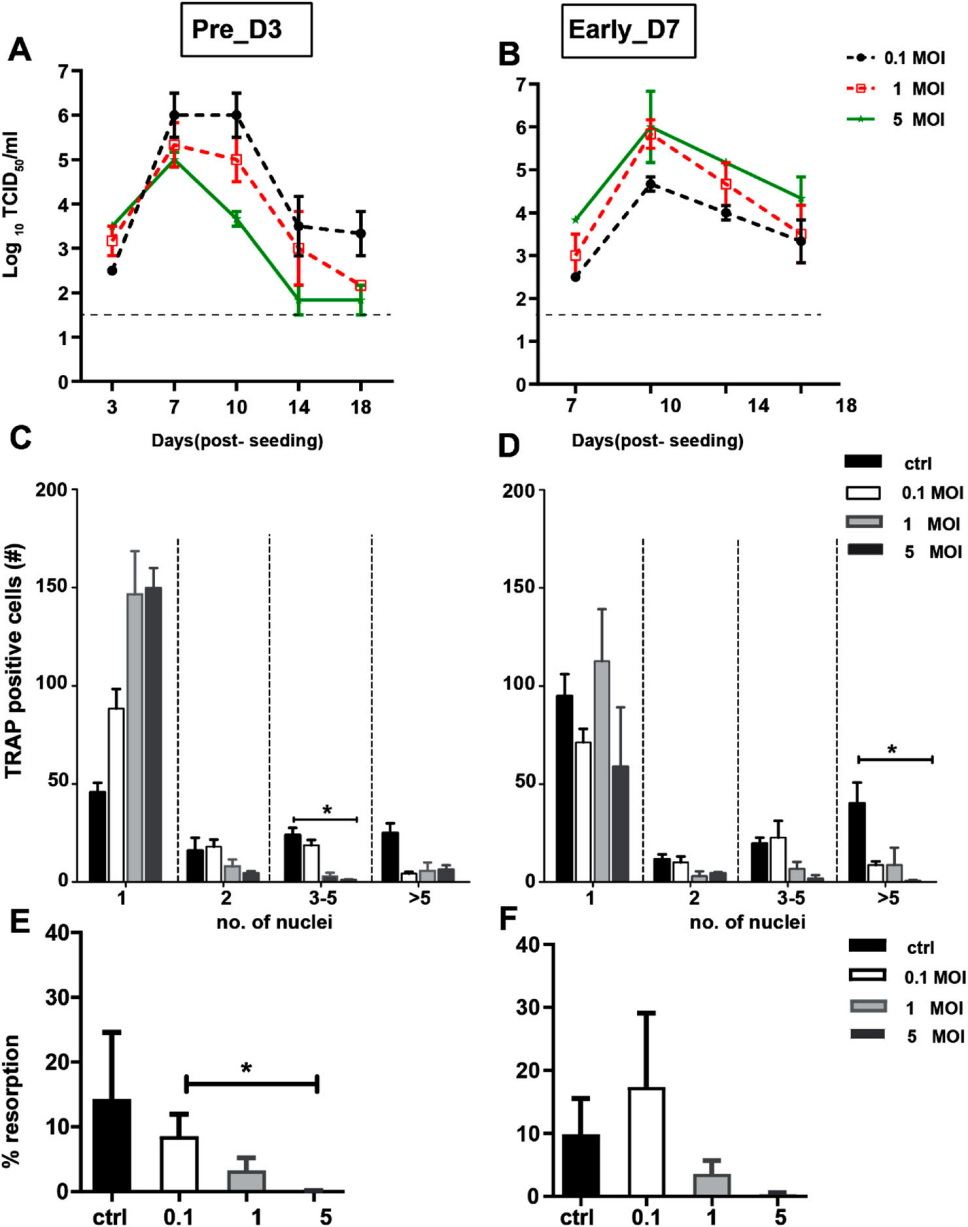
Dose dependent effect of ZIKV infection in pre and early differentiating OCs. (**A-B**) Growth curve kinetics of ZIKV infection in (**A**) pre OCs (Inf_d3) and (**B**) early differentiating (Inf_d7) using moi of 0.1, 1 and 5. (**C-D**) At moi of 1 and 5, ZIKV infection significantly reduced the formation of multinucleated OCs in both **(C)** pre- and **(D)** early differentiating OCs compared to infection using low moi (0.1). (**E-F**) At moi of 1 and 5, resorption function was also reduced in both (**E**) pre- and (**F**) early differentiating OCs compared to infection using low moi (0.1). *N* = 3 for replication kinetics, *N* = 5 for TRAP assay, *N* = 4 for resorption assay. Error bars represent SEM. * *p* < 0.05.

To understand the permissiveness and susceptibility to different viral doses, we infected pre- and early differentiating OCs with different MOI of ZIKV (0.1, 1 & 5). We selected pre-and early differentiating OCs as we observed a significant effect on osteoclastogenesis with high MOI at these stages. Growth kinetics of viral replication was similar irrespective of the viral dose (Figure 4A and B). Interestingly, infection with a low MOI in pre-OCs showed a prolonged shedding of virions compared to infection with high MOI (Figure 4A and B, respectively). The phenotypic effect of ZIKV infection at different MOI was also evaluated. While infection at low MOI resulted in a reduced number of OCs with 5 or more nuclei, we still observed OCs with 3–5 nuclei (Figure 4C and D), which indicates that infection at low MOI did not completely abolish the OC fusion compared to infection at high MOI. In addition, we observed a dose-dependent effect on the resorption capacity of ZIKV-infected pre-and early OCs showing a considerable reduction in mineral resorption at MOI of 5 (Figure 4E and D). Interestingly, in ZIKV-infected early differentiating OCs, infection with low MOI (0.1) did not affect the resorption ability of infected OCs compared to mock-infected controls (Figure 4F).

### Antibody dependent enhancement (ADE) of ZIKV infection in osteoclast precursors

**Figure 5.**
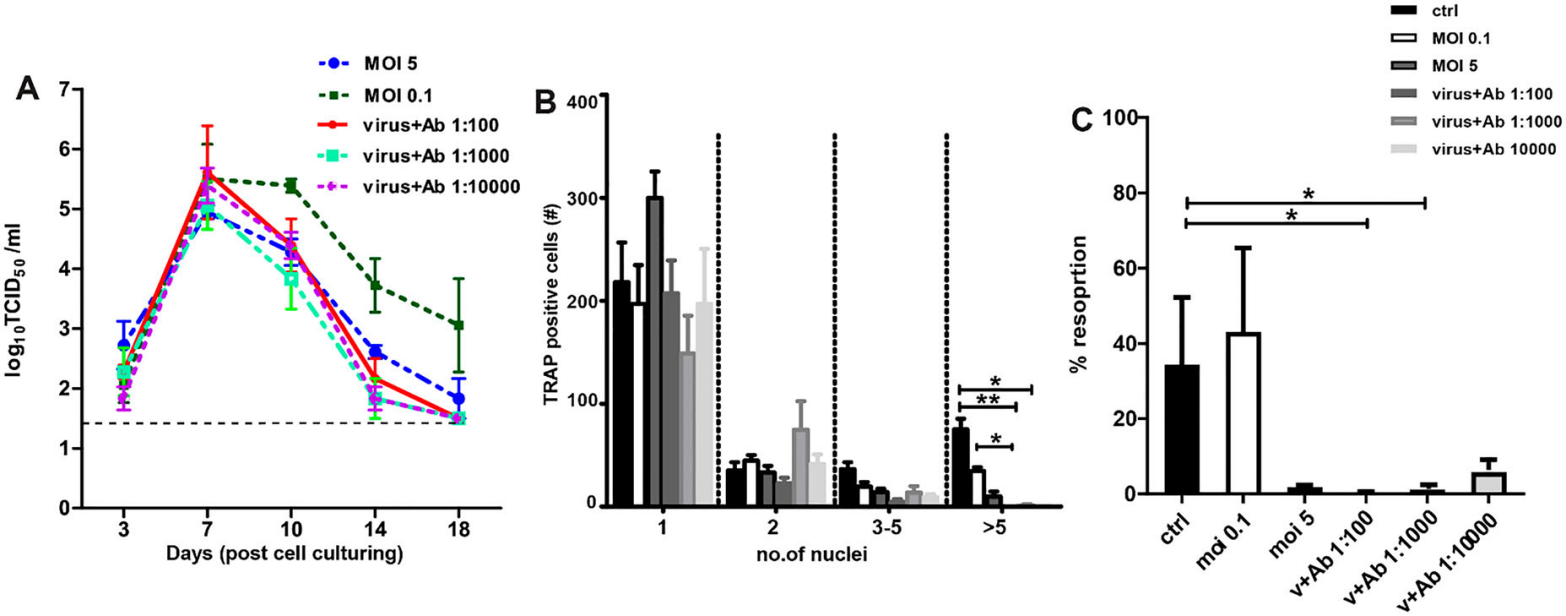
Antibody-dependent enhancement (ADE) of ZIKV infection. ADE using low moi (0.1) in the presence of pan-flavivirus cross reacting antibody in pre-OCs. (**A**) Growth curve kinetics of ZIKV infection in pre-OCs with high moi (5), and low moi (0.1) with/without antibody. (**B**) Infection at moi 0.1-ADE inhibited the formation of mature OCs identical to infection with moi of 5-without cross reacting antibody. (**C**) Infection with ZIKV MOI of 0.1 in combination with ADE also inhibited the resorption function in ZIKV-infected pre-OCs similar to infection with high ZIKV moi (5) alone. *N* = 3 for replication kinetics, *N* = 5 for TRAP assay, *N* = 4 for resorption assay. Error bars represent SEM. * *p* < 0.05.

ZIKV can infect antigen presenting cells that possess Fcγ receptors through a process called ADE in which enhancement of infection due to flavivirus cross-reactive antibodies can occur. ADE at a low MOI with a related flavivirus, DENV, has previously been shown to mimic the effects of infection with high MOI in primary human macrophages [33]. Since OCs are known to express these Fcγ receptors, we tested whether ADE at low ZIKV MOI (0.1) combined with flavivirus cross-reacting antibodies could mimic the effects seen at high ZIKV MOI alone. Intriguingly, while ZIKV infection of pre-OCs at a low MOI did not result in a reduction of bone resorption, infection with MOI 0.1 in the presence of ADE antibodies mimicked the effect of infection at high MOI without cross-reacting antibodies (Figure 5A). The effect of ZIKV infection enhancement was determined via standard TRAP assay and von Kossa staining. In conclusion, we discovered a significant reduction in the number of mature multinucleated OCs, which was recapitulated in terms of resorption ability (Figure 5B and C).

### Fusion proteins kinetics in ZIKV-infected OCs

**Figure 6.**
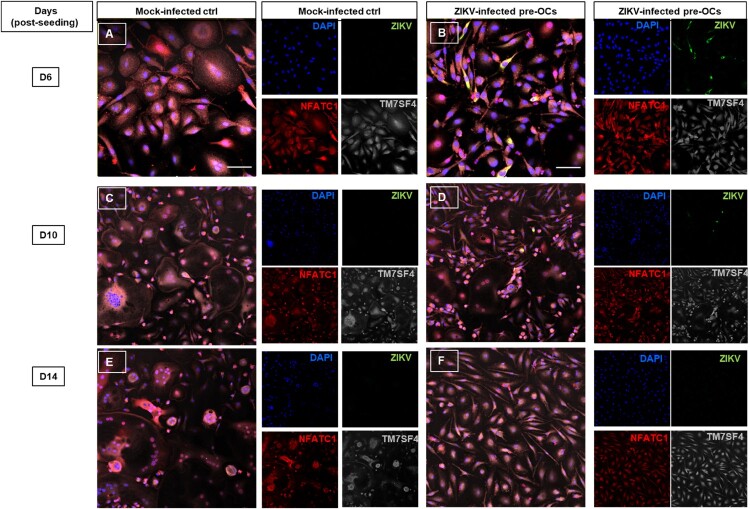
Immunofluorescence staining of ZIKV infected pre-OCs for NFATC1 and TM7SF4/DC-STAMP. Pre-OCS were infected with ZIKV (moi = 5) on day 3 post seeding. At the indicated time post cell culturing, ZIKV infected and mock infected cells were fixed, permeabilized and stained for ZIKV (green), NFACT1 (red), TM7SF4/ DC-STAMP (grey) and nuclei (DAPI, blue) for selected time points day 6 (**A-B**), day 10 (**C-D**) and day 14 (**E-F**) post seeding. The composite images are assembled based on the adjacent individual staining.

Since the effect of ZIKV on OC differentiation and function was only observed at early stage of differentiation, we hypothesized that infection at early stage affected the fusion protein kinetics, which are responsible for cell fusion to form mature OCs. Therefore, to determine the expression and kinetics of fusion-related markers, we performed IFA for nuclear factor of activated T cells cytoplasmic 1 (NFATC1), transmembrane 7 superfamily, member 4 (TM7SF4; also known as dendritic cell-specific transmembrane protein; DC-STAMP) and ZIKV itself in ZIKV-versus mock-infected (Figure 6A–D). We detected expression of both differentiation and fusigenic markers in infected and control cultures at different time points (Figure 6A–D). During late differentiation (day 14) the nuclear translocation of NFATC1 was prominent in mock-infected controls with multinucleated cells whereas in infected cultures NFATC1 expression is observed in cytoplasmic (Figure 6E and F). To determine the pattern of kinetics over the culture period, we also performed semi-quantitative analysis by measuring the mean fluorescent intensity of both markers using Image J software. Interestingly, in ZIKV-infected cultures at day 6 and 10 post-seeding, we found a reduction in the mean fluorescent intensity (MFI) for NFATC1 expression compared to mock-infected controls (*p* = 0.02) (supplementary Figure 2A). In the case of TM7SF4, similar to NFATC1, we observed differences in expression kinetics at day 6 and 10 post seeding (supplementary Figure 2B).

### ZIKV-infected pre-OCs display destabilized actin organization and actin ring structure

**Figure 7.**
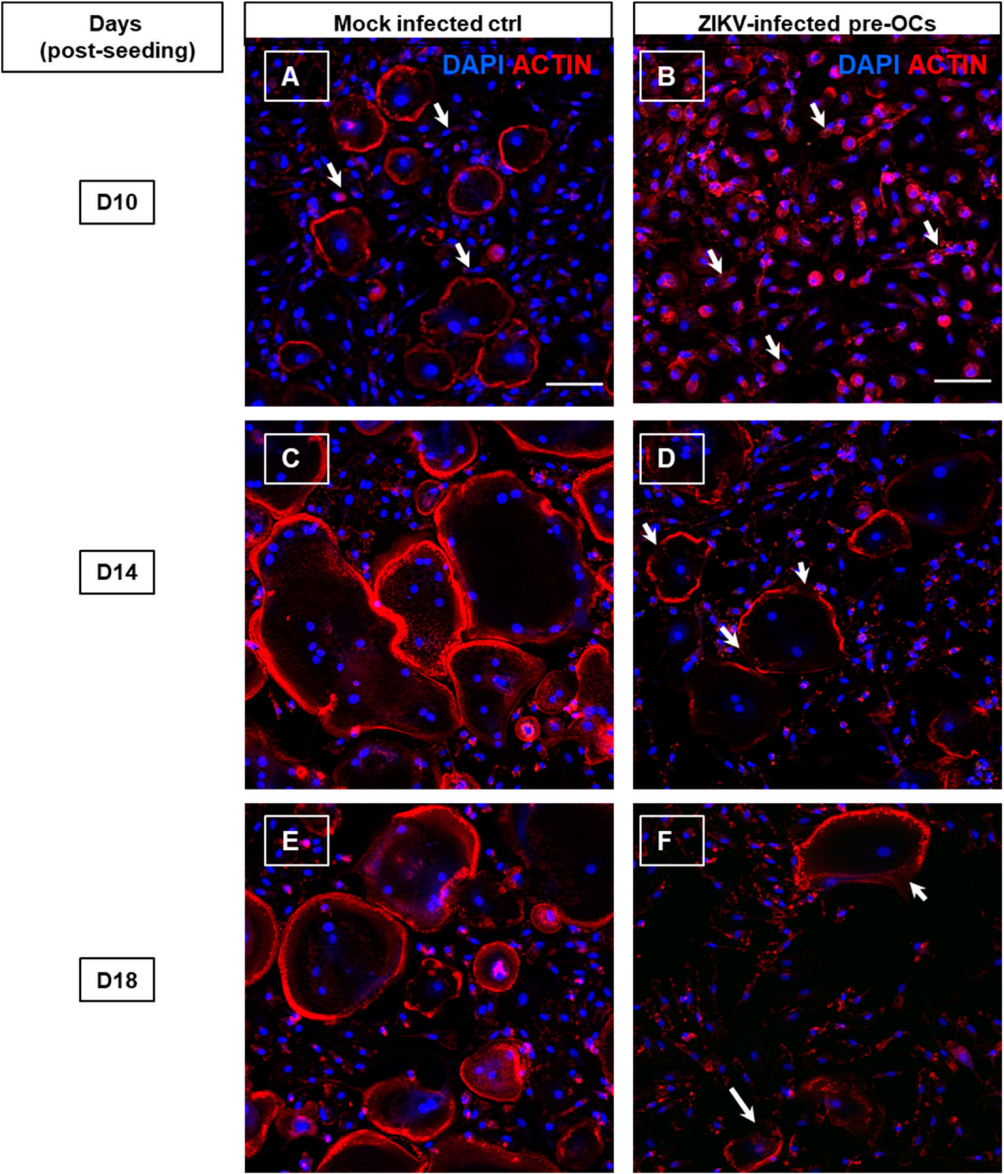
Immunofluorescence staining of ZIKV infected pre-OCs actin dynamics. Pre-OCS were infected with ZIKV (moi = 5) on day 3 post cell culturing. At the indicated time post cell culturing, ZIKV infected and mock infected cells were fixed, permeabilized and stained for actin with rhodamine-conjugated phalloidin (red) and nuclei (DAPI, blue) for selected time points such as day 10 (**A-B**), day 14 (**C-D**) and day 18 (**E-F**) post cell culturing. The arrows in the images indicate the actin organization in cytoplasm in the mononucleated OCs in (**A**) mock- and (**B**) ZIKV infected pre-OCs. (**D, F**) Arrows indicate the breaks in the actin rings towards the periphery of ZIKV infected pre-OCs.

To investigate the effect of infection on actin organization and ring formation which is essential for resorption function, we performed rhodamine-conjugated phalloidin staining in ZIKV infected and mock-infected pre-OCs for selected time points (Figure 7). Intriguingly, at day 10 post-seeding, infected cultures revealed disrupted actin organization compared to mock-infected controls (Figure 7A and B). At later time points on day 14 and 18 post-seeding, the infected cultures appeared to have fewer actin rings compared to controls, which is in line with results from the TRAP staining on day 14 (Figure 7C–F). Moreover, infected pre-OCs had more discontinuous actin rings compared to controls.

### Gene expression profile of key differentiation markers

To evaluate the effect of infection on expression levels of OC differentiation markers, qPCR was performed on multiple time points during differentiation. Although ZIKV infection affected the differentiation of pre-OCs, the gene expression profile of key differentiation markers failed to show significant differences between mock- and ZIKV-infected cells (supplementary Figure 3).

## Discussion

In the current study, we demonstrate that ZIKV can infect and replicate in human osteoclasts, which can result in a reduction of osteoclastogenesis and bone resorption. In addition, we observed that this effect is dependent on the OC differentiation stage and a high virus dose.

Except for DENV, there are limited studies on the direct effect of flaviviruses infection on OC susceptibility and differentiation [34]. Here we show that ZIKV infection induced morphological changes in infected pre-OCs. On day 3 post-infection, we observed elongated fibroblast-like cells in ZIKV-infected pre-OCs, which persisted over the culture period. These elongated cells resemble the M2 phenotype of macrophages [35], and Asian lineage ZIKV infection is shown to mediate M2-skewed immunosuppression in ZIKV-infected symptomatic patients [36]. Biophysical cues in the local microenvironment, such as microbial products, damaged cells, and activated lymphocytes, can regulate the polarization and induce phenotypic changes in monocyte-derived macrophages [37]. ZIKV-induced morphological changes might have significantly inhibited the formation of multinucleated mature OCs in infected cultures. The observed osteoclast phenotype following ZIKV infection is distinct from that described for other arboviruses, such as CHIKV and RRV, where no morphological changes were reported [38,39].

In the case of RRV and CHIKV infections, *in vivo* studies have demonstrated increased osteoclastogenesis by the indirect effect of infection [38–41]. These viruses mainly target bone forming osteoblasts for replication, which subsequently trigger osteoclastogenesis via upregulation of paracrine factors [40]. RRV, CHIKV, and DENV infected patients are known to develop rheumatic disorders, including inflammation of musculoskeletal tissue, that is mainly caused by activation of osteoclast-facilitated bone resorption and suppression of osteoblasts-mediated bone formation, collectively resulting in bone loss [23,24,38,42]. However, in the current study, we observed a direct effect of ZIKV infection on pre-OCs and concomitantly reduced osteoclastogenesis. Direct infection of osteoclasts had also been studied for human immunodeficiency virus (HIV), but unlike ZIKV infection, HIV infection led to increased OC formation [43,44]. HIV infection induced the formation of larger OCs and enhanced the mRNA expression of OCs markers, such as *TRAP*, cathepsin K, and the calcitonin receptor [CTR] [43].

We hypothesized that reduced osteoclast function due to ZIKV infection could be one possible mechanism for ZIKV-associated microcephaly. While ZIKV-induced microcephaly is shown to be primarily caused by reduced neuronal development [45,46], secondary microcephaly as a result of craniosynostosis has been reported as well [47–49]. Craniosynostosis may be the consequence of imbalances during skull development by the increased bone formation and reduced bone resorption. Defects in OC activity with excessive deposition of immature bone are known to mechanistically underlie a genetically determined bone disease called osteopetrosis, which can subsequently result in microcephaly [50]. Therefore, to understand the various skeletal aspects of ZIKV pathogenesis, the role of osteoclasts in ZIKV infection requires further investigation.

Currently, we report on a differentiation-dependent effect of ZIKV infection on osteoclastogenesis. ZIKV infection at day 10 post-seeding did not influence the formation of mature OCs while infection at early stage of differentiation had shown a pronounced inhibitory effect on osteoclastogenesis. This temporal effect of infection is also observed in a previously reported study for an an-aerobic bacterium, where haemoglobin receptor protein (HbR) of Porphyromonas gingivalis has an inhibitory effect on OC formation and activity during the first 24 h of culture [51]. The temporal inhibitory effect could be due to the influence on RANKL-mediated differentiation markers or specific targets of pre-OCs, which play a crucial role in the early stages of OC differentiation.

In addition to having direct effects on osteoclast formation and activity, ZIKV may employ ADE mechanisms to infect OCs. ADE is mainly mediated by Fcγ receptors, which are distributed on OCs, therefore enabling ADE to serve as an added mechanism for enhanced ZIKV pathogenesis combined with cross-reacting flavivirus antibodies. As ZIKV and DENV geographically overlap around the globe, the risk associated with flavivirus background immunity cannot be ignored. In the current study, we observed that ZIKV infection at low MOI (0.1) did not affect the function of OCs at an early stage of differentiation, but in combination with DENV-derived cross-reacting antibodies it mimicked the effect on osteoclastogenesis as seen with a high ZIKV MOI [5]. Similar findings have previously been reported for DENV in primary human macrophages, where low MOI in combination with ADE mimicked the infection with high MOI in elevating the fusion activity of DENV [33], thus providing a benefit for infection efficiency. This suggests a potential risk for enhanced ZIKV infection in OCs (bone tissue macrophages), particularly in flavivirus endemic areas, and may explain, in part, why ZIKV infection has been associated with bone pathologies in these flavivirus endemic areas.

In symptomatic ZIKV infection, arthralgia or rheumatic complaints were frequently reported during the ZIKV epidemic [4,5], but fewer efforts have been put forward to explore the underlying factors behind ZIKV-induced arthralgia. For other arthritogenic viruses, such as RRV and CHIKV, inflammatory bone loss due to increased osteoclast activity is the leading cause of disturbed bone remodeling [13]. To maintain bone homeostasis (bone remodeling), the balanced activity of osteoblasts and osteoclast is essential, and a disturbing function of either cell type can induce bone-related pathologies [52]. In the case of ZIKV infection, we have previously shown that early ZIKV infection perturbs the function of bone-forming osteoblasts, which are very essential in determining the pathogenesis of the rheumatic disease. Therefore, to extend the role of osteoclasts and understand the pathogenesis of ZIKV induced rheumatic disorder, it will be essential to study osteoblast-osteoclast interaction in primary co-culture models.

ZIKV-infected OCs exhibited impeded OC formation with a concomitant reduction of the fusion index. OC multinucleation results from the cell– cell fusion of mononuclear osteoclasts and is the most important characteristic of OCs, which involves a sequence of events [53]. RANK and RANKL are key regulatory molecules for the formation and activation of OCs. Transcription factors such as NF-κB, c-FOS, and NFATC1 are downstream factors of RANKL-mediated activated pathways, being essential for osteoclast differentiation and in turn regulating OC fusion mainly via TM7SF4/DC-STAMP [54,55]. Based on the significance of fusion proteins in osteoclast formation, we hypothesized that a reduced fusion index and inhibition of OC formation might be due to the inhibition of NFATC1 activation (i.e. nuclear translocation), which in turn could affect DC-STAMP expression. Indeed, we observed that nuclear translocation of NFATC1 in ZIKV-infected pre-OCs was inhibited compared to mock-infected controls. However, this is contrary to previous findings for DENV, where infection-mediated NFATC1 translocation is reported in DENV-infected OCs [34]. We showed a reduction in MFI of NFATC1 in ZIKV-infected pre-OCs, mainly during early time points of differentiation, which might be essential in determining the overall phenotype of infected cultures. To better understand the interplay of differentiation and fusion factors following ZIKV infection, future studies are warranted.

In addition to fusion, the adhesion property of OCs is also very important to eventually perform its resorption activity [56]. In this study, we demonstrated a reduction in the adhesion ability of ZIKV-infected OCs compared to those of mock-infected controls. For resorption function, the tight adhesion of OCs is indispensable. Actin organization is critical for the overall binding of the OCs and the establishment of the sealing zone to allow for the resorption of both organic and inorganic bone substances. In general, activation of integrin receptor complexes induces the re-organization of the actin cytoskeleton [57,58]. In the current study, ZIKV infected pre-OCs exhibited an altered actin organization compared to controls, which most likely influences the actin ring formation in later stages as exemplified by a notable reduction of the number of actin rings but also more frequent discontinuation of the rings, which implicates disturbed resorption activity. A previously published study described similar findings that disrupted actin dynamics inhibited overall osteoclastogenesis [59], but in the case of arboviruses, to our knowledge, ZIKV is the first arbovirus described to cause the actin ring disturbance in osteoclasts.

In the current study, there are a few potential limitations. Firstly, we have performed experiments mainly by deriving PBMCs from one donor. Although there is a possibility of having subtle differences between PBMC donors, our reinforcing observations using multiple assays demonstrates the consistent inhibitory effect of ZIKV infection by following high moi and low moi-ADE. Secondly, our current model is an isolated culture system which is very useful to screen for direct effects of ZIKV infection on the osteoclast phenotype, but ultimately these findings need further validation in *in vivo* models. Thirdly, the phenotypic effects observed are not supported by the gene expression profiles, which can be partly explained due to choice of time point, having to deal with a heterogeneous cell population and concomitant expression of key markers. Investigating these markers and their interactions at the protein level will be the focus of our future studies.

In conclusion, we have developed and characterized a new primary *in vitro* model to study the role of osteoclastogenesis in ZIKV pathogenesis. Using this model, we showed that ZIKV infection perturbs osteoclast differentiation and function, and hypothesized an alternative mechanism by which ZIKV can induce bone pathology, resulting in microcephaly. This model will help to identify novel skeletal targets to develop therapeutic and preventive measures against ZIKV.

## Supplementary Material

Supplemental Material

## Data Availability

The data that support the findings of this study are available from the corresponding author [B.R,& B.C.J.E], upon reasonable request.
