# Supplementary material for: Zika virus infects human osteoclasts and blocks differentiation and bone resorption
Source: Emerg Microbes Infect. 2022 Jun 14;11(1):1621–34. doi: 10.1080/22221751.2022.2086069 (PMC9225750; doi:10.1080/22221751.2022.2086069)
Supplement: Supplemental Material [file TEMI_A_2086069_SM8915.docx]

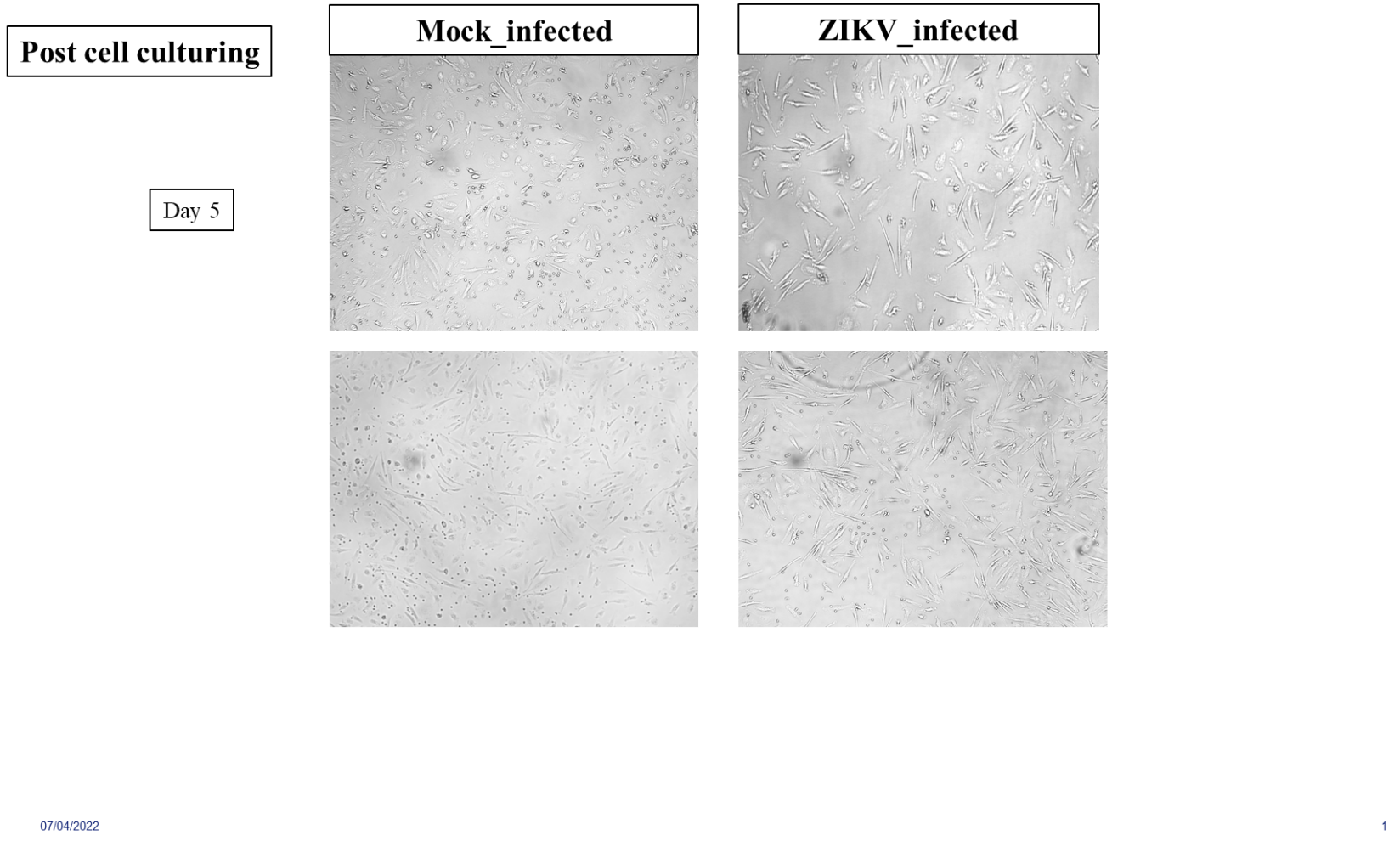


**Supplementary figure 1**: Bright field images of mock and ZIKV infected cells at day 5 post cell culturing.


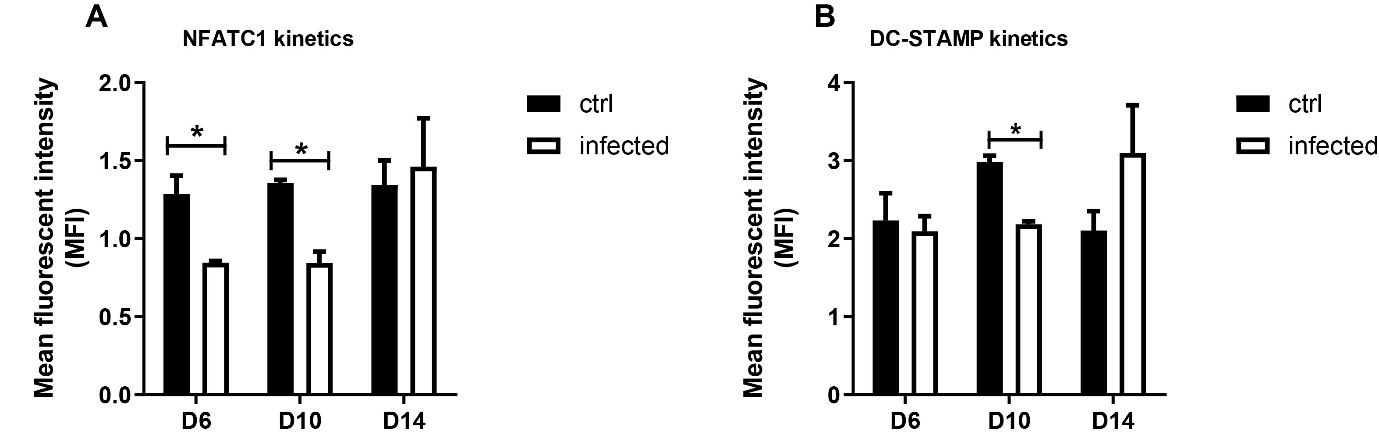


**Supplementary figure 2**. Intracellular mean fluorescent intensity (MFI) values of NFATC1


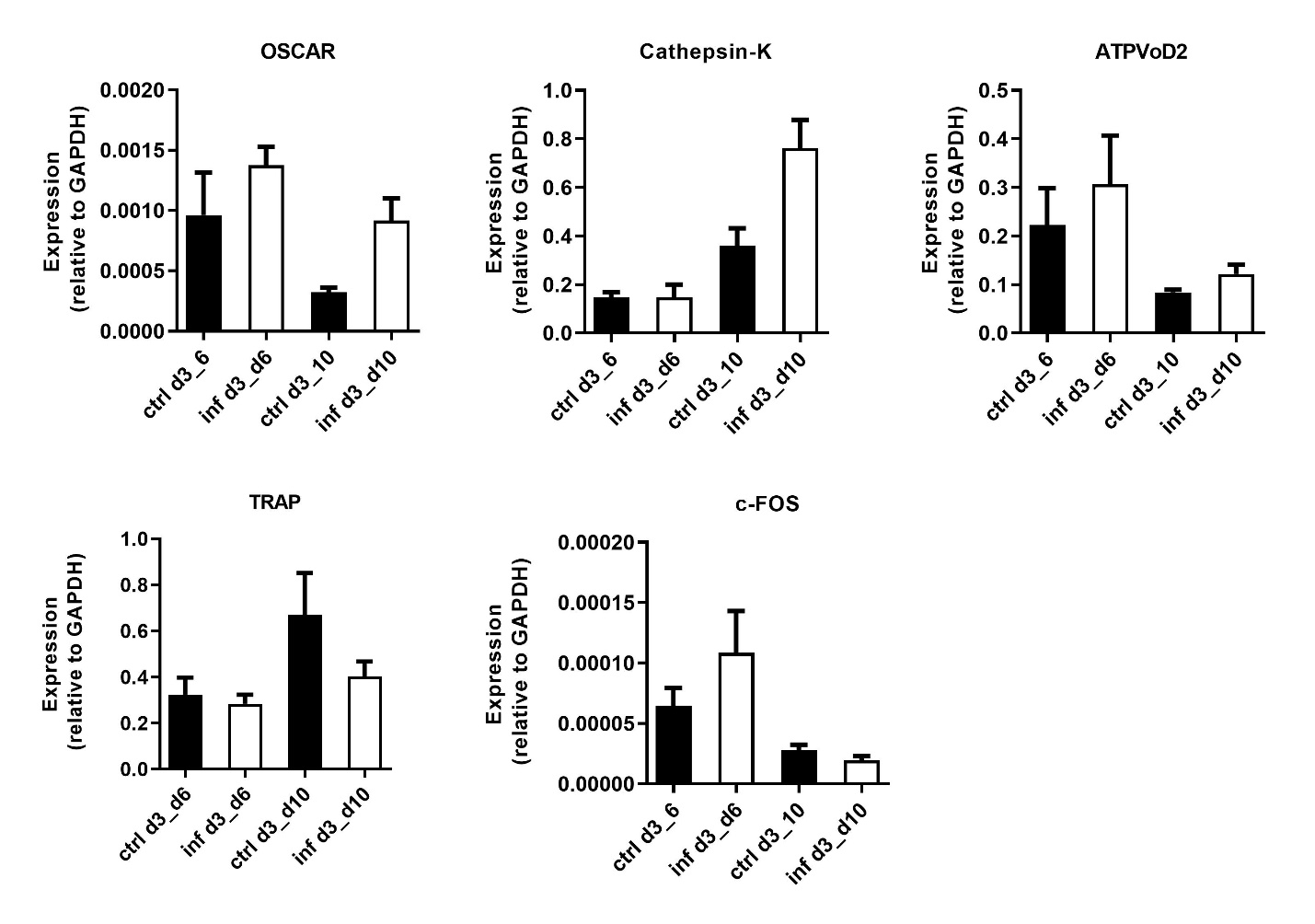


**D E C**

**A B C C**

**Supplementary figure 3**: mRNA expression kinetics of key osteoclast differentiation markers.
